# Treatment of Deep Vein Thrombosis with Continuous IV Infusion of LMWH: A Retrospective Study in 32 Children

**DOI:** 10.1155/2011/981497

**Published:** 2011-04-14

**Authors:** Veronika Fiamoli, Jan Blatny, Ondrej Zapletal, Svetlana Kohlerova, Eva Janousova

**Affiliations:** ^1^Department of Clinical Haematology, Centre for Thrombosis and Haemostasis. Children's University Hospital, Masaryk University, 62500 Brno, Czech Republic; ^2^Institute of Biostatistics and Analyses, Masaryk University, 62500 Brno, Czech Republic

## Abstract

Thirty-two consecutive children aged 0–18 years with VTE treated with LMWH administered as a continuous infusion (CI) were identified at the Children's University Hospital Brno. The treatment led to at least partial resolution of the thrombus within two weeks in 85% of patients. There were no adverse events or increased bleeding reported in any patients. No recurrences were observed during a followup period of 6 months. Although continuous infusion should not replace subcutaneous (SC) administration of LMWH, CI appeared to be safe and efficient and may provide an alternate method of administering LMWH in a subset of the paediatric population where SC administration may not be feasible. Further prospective studies are needed to support the promising findings of our pilot clinical observation.

## 1. Introduction

The incidence of VTE-venous thromboembolism (deep vein thrombosis, pulmonary embolism) is age dependent with the lowest risk occurring in children [1–3]. The estimated incidence of deep vein thrombosis (DVT) and pulmonary embolism (PE) in the general paediatric population is 0.07/10 000 and 5.3/10 000 among hospital admissions, respectively [4–7]. In the last decade, however, the incidence of VTE in children appears to be increasing probably because of increased awareness of the problem and improved diagnostic methods [8, 9]. DVT/PE develops in children often as a secondary complication of other underlying disorders. These prothrombotic risk factors can be either inherited or acquired. Inherited prothrombotic risk factors are responsible for only 5–7% of VTE in children [6, 7]. Idiopathic VTE occurs in less than 1% of newborns, and in less than 5% of children compared to approximately 40% of adults [6, 7, 10, 11]. Children less than one year of age and teenagers appear to be at a higher risk for developing secondary VTE [6, 10–12]. 

Low molecular weight heparins (LMWHs) are currently the standard of treatment for DVT in children for its more predictable pharmacokinetics and better bioavailability than unfractionated heparin [13]. It is also associated with lower risk of heparin-induced thrombocytopenia (HIT), bleeding, and osteoporosis [14]. Other alternatives include, coumarins, pentasaccharides, and under specific circumstances, thrombolytics. The standard method of LMWH administration is via the subcutaneous (SC) route. It enables home treatment, and in the vast majority of patients, it does not need frequent monitoring once a therapeutic and safe level has been achieved. However, there may be a subset of children, in whom the benefits of SC administration may be outweighed by the potential risks and difficulties related to repeated SC injections. The aim of this pilot project was to evaluate the safety and efficacy of continuous infusion (CI) of LMWH in those children with a DVT who already had a central venous access device in place for other reasons.

## 2. Methods

Between January 2003 and June 2009, 32 consecutive children with a DVT who were treated with LMWH administered as a CI were identified at the Children's University Hospital, Brno. Deep vein thrombosis was diagnosed with Doppler ultrasound (Siemens, type Antarez, linear probe VFX 15-3, 10 MHZ), increased D-dimer levels (STA Liatest D-Di, Diagnostica STAGO, France) and by clinical symptoms and signs that included: tenderness, pain, and swelling at the site of thrombosis, where applicable.

Based on our previous preliminary study, the starting dose of LMWH in all our patients was 240 IU/kg/24 hrs regardless of age and/or thrombosis site [15]. Nadroparin and Dalteparin were the two routinely used LMWHs at the Children's University Hospital. LMWH was administered by continuous infusion through an intravenous pump, and the infusion set was changed every 8 hours, with doses being administered as 80 IU/kg/8 h diluted in normal saline. Rate of the infusion was 1 mL/hr. Further dosing was tailored individually for each patient to reach the required therapeutic anti-Xa level. Blood samples for anti-Xa level measurements were taken twelve hours after initiation of infusion and after any dose adjustments or change in infusion rates. The dose of LMWH was adjusted to keep anti-Xa levels between (0.5–1.0 IU/mL). Continuous infusion was not interrupted for more than 2 hours at any one time, and only in exceptional situations, such as Doppler ultrasonography evaluation when the patient had to be transported to the radiology suite from the inpatient ward. 

The efficacy of treatment was assessed by the improvement in clinical symptoms as evaluated during regular ward rounds, as well as by repeated Doppler ultrasonography (week 1, week 2 and week 6 after the diagnosis of the thrombosis) along with quantitative D dimer testing. Anti-Xa levels (Coamatic Heparin, Chromogenix, Italy) were monitored daily at least during the first five days of treatment, and then at frequent intervals depending on the individual subject. The aim was to achieve a stable anti-Xa level. 

 Platelet counts were monitored daily, especially in patients with low platelets. None of the patients had any renal disorders. Liver function tests were not monitored routinely in our patients. Therapy with CI of LMWH was stopped when recanalization of the vein had been achieved or the thrombus had organized and unchanged on two consecutive Doppler ultrasound examinations. Patients were than continued on therapy with either SC LMWH or switched to vitamin K antagonists (VKA) treatment for at least 3 months, and then were followed for at least six months to monitor for any recurrence. The safety of the treatment was clinically assessed by nurses who recorded any bleeding or other possible adverse events related to the CI of LMWH. 

Descriptive statistics, such as mean, median, minimum and maximum values and interquartile range (IQR), were used for description and comparison of duration of treatment, dose of LMWH. and level of anti-Xa. 

The study was approved by the local Ethics Committee.

## 3. Results

**Figure 1:**
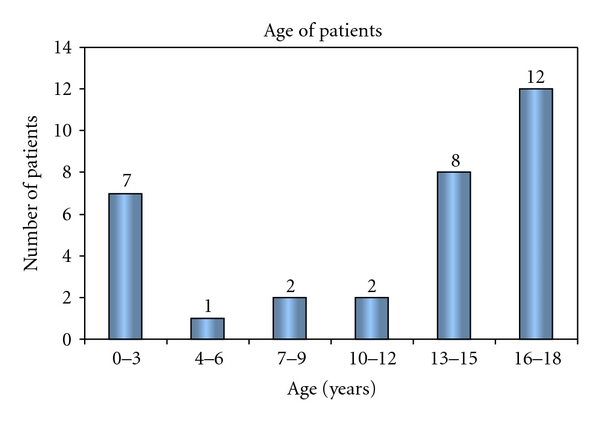
Number of patients according to age.

**Figure 2:**
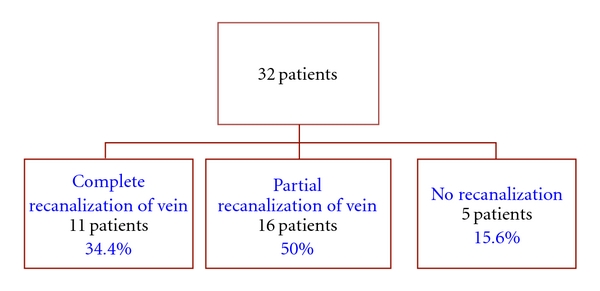
Thrombus resolution.

There were 18 (56%) males and 14 (44%) females. Their ages at the time of treatment varied from newborns to 18 years, with median of 14 years (IQR 7–16 years). (Figure 1). Site of thrombosis included CVL-related thrombosis (*n* = 9), DVT of lower extremity veins (*n* = 11), DVT of upper extremity veins (*n* = 3), lower vena cava thrombosis (*n* = 3), sino-venous thrombosis (*n* = 4), and pulmonary embolism (*n* = 2). CI of LMWH was tolerated well by our patients. There were no adverse events or increased bleeding reported in any of these patients during CI of LMWH. This therapy led to complete, recanalization of the affected vein in 11 (34.4%) patients and partial recanalization in 16 (50%) patients. In 5 (15.6%) patients recanalization was not evident despite therapeutic levels of anti-Xa (Figure 2). 

The median of duration of treatment with CI LMWH was 13.5 days (IQR 9.5–22.0 days). The median dose of LMWH administered by continuous infusion to reach required therapeutic anti-Xa levels was 240 IU/kg/24 h (IQR 220–250 IU/kg/24 h). The median anti-Xa level achieved on this protocol was 0.54 IU/mL (IQR 0.43–0.69 IU/mL) (Table 1). During the 6 months of followup, no recurrences were observed in this cohort of patients.

## 4. Discussion

The aim of this pilot retrospective clinical observational study was to evaluate an alternate strategy of administering anticoagulant therapy in the treatment of thrombosis in children. The standard treatment for DVT is by SC administration of LMWH. These recommendations are derived from the REVIVE study, which has shown that LMWH when administered SC appeared to be safe and effective for use in children with DVT [13, 14]. 

Based on our own previously published clinical data [15], we used a dose that was found to be safe and efficacious in initiating the CI. This study corroborates our early findings about the efficacy and safety of treatment with CI of LMWH. CI with LMWH resulted in at least partial to complete resolution of the thrombus in 84.6% of children. This compares favourably with that of SC administration as published in the literature [16, 17]. Anti-Xa levels achieved with CI were similar to those achieved with SC administration. It is to be noted that the level of anti-Xa assessed during SC treatment reflects the maximal peak anti-Xa activity after bolus SC injection. On the other hand, levels of anti-Xa during CI aim to reflect the stable steady state of anti-Xa activity in patients' plasma. Thus, information about anti-Xa gained during CI of LMWH may not be comparable to that of SC administration. Further studies to delineate the pharmacokinetics of LMWH during CI administration will be needed to help understand the significance of these levels and tailor them with that of efficacy. In contrast to the REVIVE study, we have not observed any adverse events in our patients. This finding is similar to the finding by Hoffmann et al. who showed that intravenous (IV) bolus administration of LMWH is as safe as SC administration and is not associated with any higher risk of adverse events [18]. 

CI of LMWH may be advantageous especially in children who cannot be treated at home, are admitted to the hospital, and already have central venous access for other reasons (ICU patients, oncology patients, or patients with burns of a significant percentage of their body surface). CI avoids repeated painful SC injections, and thus, may improve a child's quality of life. In children with very low weight, malnourished or those who lack subcutaneous fat, it may be difficult to give a precise dose via the SC route due to the very small amount of medication injected. Furthermore, in very small children and neonates, repeated SC injections may be limited by body surface available for administration. When administered by CI, LMWH has a shorter half-life and this would be beneficial in patients with considerable risk of bleeding, for example, patients with chemotherapy induced-thrombocytopenia. In addition, these oncology patients have increased burdens and hassles associated with the treatment of their disease and minimizing invasive interventions could perhaps increase their quality of life.

## 5. Conclusion

In our patients, the treatment of VTE with LMWH by continuous infusion was estimated to be safe and efficient and may provide an alternate method of administering anticoagulation in the paediatric population. Further prospective studies are needed to support the promising findings of our pilot clinical observation.

## Figures and Tables

**Table 1:** Treatment with continuous infusion of LMWH.

	*N*	Mean	Median	IQR	Min-max
Duration of the treatment with continuous infusion of LMWH (in days).	32	16.5	13.5	9.5–22.0	5.0–44.0
Weighted dose of LMWH during the treatment with CI LMWH (in IU/kg/d).	58	244.5	240.0	220.0–250.0	200.0–330.0
Summarized level of anti-Xa during treatment with CI LMWH (in IU/mL).	103	0.58	0.54	0.43–0.69	0.13–1.41

*IQR indicates interquartile range.
